# Persian Gulf Bioactive Natural Drugs

**DOI:** 10.17795/jjnpp-19354

**Published:** 2014-04-07

**Authors:** Mohammad Reza Shushizadeh

**Affiliations:** 1 Marine Pharmaceutical Science Research Center, Ahvaz Jundishapur University of Medical Sciences, Ahvaz, IR Iran

**Keywords:** Persial Gulf, Aquatic Organisms, Biological Products

Oceans cover 70% of the our planet surface. The Persian Gulf, which is a shallow marginal sea of the Indian Ocean and bordered on the west by the Arabian Precambrian shield, is a favorable habitat for aquatic animals, plants, and microorganisms (1). Natural products are chemical compounds made by these organisms. Natural products are often called secondary metabolites because they are chemical materials that are not essential for life. Primary metabolites are those compounds that are essential for life of the organism such as growth, development, and reproduction (2). The compounds produced by or found in marine organisms such as soft corals, sponges, algae, mollusks, phytoplanktons, tunicates (ascidians), echinoderms, and bacteria have been shown to have a wide variety of applications as pharmaceuticals products for humans or other animals. They are used as antibacterial, analgesic, anti-inflammatory, antimalarial, anticancer, antiparasitic, and antiviral agents. Although large numbers of novel compounds have been isolated from marine organisms and many of these substances have pronounced biological activity, only very few have been marketed as pharmaceutical products (3).

Progress in this branch is achieved by observation of biological phenomena, isolation and structure elucidation of the key compounds by chromatographic and spectroscopic methods, and biosynthesis of the natural products and their bioassays. Several new companies have focused on the discovery of more effective drugs based on natural products of marine microorganisms. In recent years, the improvement of isolation technologies has yielded a considerable number of potential new drug and other metabolites from microorganisms of marine ecosystems (4). Hence, marine natural products represent a valuable foundation for the discovery of novel biologically active compounds. The potential therapeutic applications provided by these molecules along with their unique structural features have encouraged substantial scientific interest and investigations.
